# Handheld digital microscope for dermatoscopy

**DOI:** 10.1016/j.jdin.2025.11.017

**Published:** 2025-11-28

**Authors:** Chenan A. Huang, Anna K. Martino, Emily E. Antalika, Megan A. Bernier, Clara H. Ambrose, Sara M. Asbeck, Steven R. Feldman, Zeynep M. Akkurt

**Affiliations:** a Department of Dermatology, Center for Dermatology Research, Wake Forest University School of Medicine, Winston-Salem, North Carolina; b Wake Forest University School of Medicine, Winston-Salem, North Carolina; c Wake Forest University, Winston-Salem, North Carolina; d Department of Dermatology, Wake Forest University School of Medicine, Winston-Salem, North Carolina; e Department of Pathology, Wake Forest University School of Medicine, Winston-Salem, North Carolina; f Department of Social Sciences & Health Policy, Wake Forest University School of Medicine, Winston-Salem, North Carolina

**Keywords:** affordability, dermatoscope, dermatoscopy, dermoscopy, diascopy, digital microscope, medical education, nevi, pigmented lesion, polarized light, USB microscope

Dermatoscopy is a valuable tool for noninvasive evaluation of skin lesions, particularly with pigmented lesions, and increases diagnostic accuracy when identifying skin cancer.^1^ However, the average cost of a high-quality dermatoscope ($300-$1800) may limit access for learners, midlevel providers, nondermatology providers, and rural/low-resource settings. In comparison, Universal Serial Bus-connected digital microscopes range from $35 to $400 and have been described for diagnosing skin lesions, but they require an inconvenient cord and external laptop.^2^ A potential solution may be handheld digital microscopes, which range from $20 to $40 and offer adequate image quality with a convenient built-in screen and memory (Supplementary Fig 1, available via Mendeley at https://data.mendeley.com/datasets/vw2tdrn24b/1). Other advantages to handheld digital microscopes include user-friendliness, simple image capture, and the ability to store pictures in a useful JPEG format.

Although digital microscopes lower the barrier to entry, they have limitations. In comparison with dermatoscopes, digital microscopes have a narrow focal length, small field-of-view, low light intensity, and no polarized light. High magnification and short focal length may result in difficulty locating small lesions. Larger lesions may require scanning to view all areas, and thick papules only the slice within focus. When combined, these specifications result in higher magnification and lower image quality than a traditional dermatoscope (Fig 1). With noncontact and nonpolarized light, superficial structures like scale, comedo-like openings, milia-like cysts, pigment networks, and vessels can be seen, allowing for the detection of actinic keratoses, seborrheic keratoses, nevi, sebaceous hyperplasia, cherry angiomas, and subungual hemorrhages (Supplementary Figs 2 and 3, available via Mendeley at https://data.mendeley.com/datasets/vw2tdrn24b/1).

**Fig 1:**
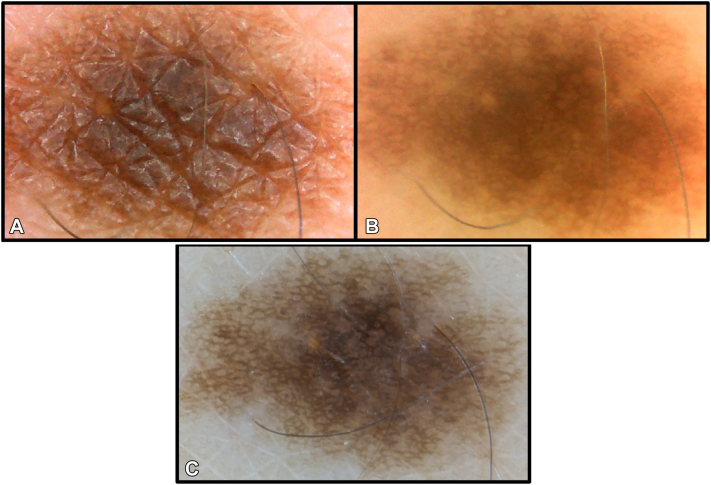
Forearm nevus. The nevus has a diameter of 2 mm. The nevus was visualized with nonpolarized light **(A)** and cross-polarization **(B)** using a NUOTON ABS-506A digital microscope. Glare is removed using cross-polarization, and the pigment network can be seen with similar quality to dermatoscopy **(C)** captured using the DE-400 Dermatoscope (Shenzhen Iboolo Optics Co). Digital microscope images **(A, B)** were cropped without zoom, and the dermatoscopy image **(C)** was cropped with zoom to improve comparison.

Two modifications—cross-polarization and diascopy—can be adapted to improve image quality. For these modifications, a handheld digital microscope with adjustable focus (NUOTON, ABS-506A, $30, Amazon.com) was used. Diascopy using glass or acrylic slides and alcohol or water can approximate nonpolarized contact dermatoscopy (Supplementary Fig 5, available via Mendeley at https://data.mendeley.com/datasets/vw2tdrn24b/1). Nonpolarized contact dermatoscopy, and thus, digital microscopes with diascopy, can more specifically diagnose lesions such as nevi and seborrheic keratoses by improving visualization of structures like milia-like cysts and comedo openings.

Polarization may be added with polarizing film (Selens, A4, US$12, Amazon.com). Polarizing sheets can be cut using a razor blade to fit various designs. Filters can be adhered in a ring-like fashion on light sources in combination with a separate circular filter covering the camera lens (Supplementary Fig 4, available via Mendeley at https://data.mendeley.com/datasets/vw2tdrn24b/1). Cross-polarization reduces light intensity, and selecting models with adjustable light intensity can mitigate the effect of light attenuation by polarization. By augmenting with cross-polarized filters, vascular components and deeper pigment networks may be visualized while surface glare is eliminated (Fig 1).^2^

Digital microscopes lower the barrier of entry for students, midlevel providers, and nondermatology providers learning dermatoscopy. Although some lesions may be more difficult to visualize with a digital microscope than a dermatoscope, the image quality is adequate to become familiar with dermatoscopic characteristics. Inexpensive modifications using polarizing filters and diascopy can further improve the utility of digital microscopes. Learners benefit from early exposure to common algorithms (ie, ABCDEs, 2-step, and triage amalgamated dermatoscopy algorithms) and familiarity with using a handheld tool for skin examinations. Better identification of benign lesions by nondermatology providers may reduce unnecessary referrals to dermatology.

## Conflicts of interest

Dr Feldman has received research, speaking and/or consulting support from Eli Lilly and Company, GlaxoSmithKline/Stiefel, AbbVie, Janssen, Alovtech, vTv Therapeutics, Bristol-Myers Squibb, Samsung, Pfizer, Alumis, Boehringer Ingelheim, Oruka, Amgen, Dermavant, Arcutis, Novartis, UCB, Helsinn, Sun Pharma, Almirall, Galderma, Leo Pharma, Mylan, Celgene, Ortho Dermatology, Menlo, Merck & Co, Qurient, Forte, Arena, Biocon, Accordant, Argenx, Sanofi, Regeneron, the National Biological Corporation, Caremark, Teladoc, BMS, Ono, Micreos, Eurofins, Informa, UpToDate, Verrica, and the National Psoriasis Foundation. He is founder and part owner of Causa Research and holds stock in Sensal Health. Drs Huang, Bernier, Asbeck, and Akkurt and Authors Martino, Antalika, and Ambrose have no conflicts of interest to declare.
